# Comparing the Effects of Anti-TNF Agent and Ustekinumab on Small Bowel Inflammation in Crohn’s Disease: Inverse Probability Weighting With Stabilized Weights of Propensity Scores

**DOI:** 10.1093/crocol/otae033

**Published:** 2024-05-14

**Authors:** Yuki Hirata, Daisuke Nishioka, Koji Nishida, Hikaru Shimizu, Noboru Mizuta, Keijiro Numa, Kei Nakazawa, Kazuki Kakimoto, Takako Miyazaki, Shiro Nakamura, Hiroki Nishikawa

**Affiliations:** Second Department of Internal Medicine, Osaka Medical and Pharmaceutical University, Takatsuki City, Osaka 569-0801, Japan; Department of Medical Statistics, Research & Development Center, Osaka Medical and Pharmaceutical University, Takatsuki City, Osaka 569-0801, Japan; Second Department of Internal Medicine, Osaka Medical and Pharmaceutical University, Takatsuki City, Osaka 569-0801, Japan; Second Department of Internal Medicine, Osaka Medical and Pharmaceutical University, Takatsuki City, Osaka 569-0801, Japan; Second Department of Internal Medicine, Osaka Medical and Pharmaceutical University, Takatsuki City, Osaka 569-0801, Japan; Second Department of Internal Medicine, Osaka Medical and Pharmaceutical University, Takatsuki City, Osaka 569-0801, Japan; Second Department of Internal Medicine, Osaka Medical and Pharmaceutical University, Takatsuki City, Osaka 569-0801, Japan; Second Department of Internal Medicine, Osaka Medical and Pharmaceutical University, Takatsuki City, Osaka 569-0801, Japan; Second Department of Internal Medicine, Osaka Medical and Pharmaceutical University, Takatsuki City, Osaka 569-0801, Japan; Second Department of Internal Medicine, Osaka Medical and Pharmaceutical University, Takatsuki City, Osaka 569-0801, Japan; Second Department of Internal Medicine, Osaka Medical and Pharmaceutical University, Takatsuki City, Osaka 569-0801, Japan

**Keywords:** Crohn’s disease, small intestine, anti-TNF-α antibody, ustekinumab

## Abstract

**Background:**

Endoscopic mucosal healing serves as a critical predictor for achieving long-term remission in Crohn’s disease treatment. Recent data indicate that the effectiveness of healing varies based on the location of gastrointestinal inflammation. Additionally, reports suggest that antitumor necrosis factor-α (anti-TNF-α) agents exhibit reduced efficacy in treating small intestinal inflammation compared to colorectal inflammation. Conversely, limited research exists regarding the impact of the anti-IL12/23 agent ustekinumab (UST) on small intestinal inflammation. This study aimed to compare the effects of anti-TNF-α agents and UST on small intestinal inflammation using propensity score analysis.

**Methods:**

This retrospective observational study involved 70 patients with Crohn’s disease who had inflammation in the small intestine and had initiated treatment with either anti-TNF agents or UST between March 2015 and August 2021. Endoscopic findings were evaluated before treatment commencement and at 1–2 years post-treatment initiation. The propensity score was employed to compare the efficacy of TNF agents and UST on small bowel inflammation.

**Results:**

Ustekinumab exhibited greater improvement in the small intestinal endoscopy score than anti-TNF-α antibodies according to the propensity score analysis (inverse probability weighting; *P* = .0448). However, no significant disparity was observed in the overall improvement of endoscopic scores between UST and anti-TNF-α antibodies (*P* = .5938).

**Conclusions:**

This study suggests that UST might be more effective than anti-TNF-α agents in treating small intestinal inflammation in Crohn’s disease.

## Graphical Abstract

**Graphical Abstract:**
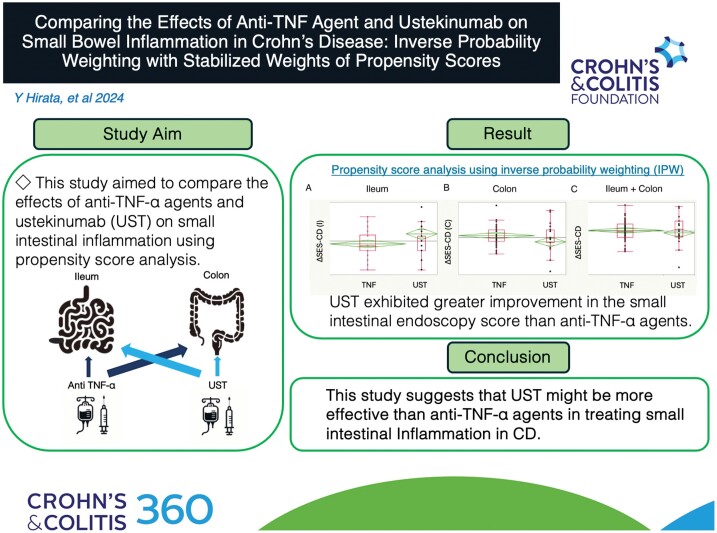

## Introduction

In Crohn’s disease, mucosal healing holds substantial significance as an endpoint for both assessing treatment effectiveness and predicting treatment outcomes.^1,2^ Furthermore, it has been demonstrated that cases exhibiting mucosal healing experience reduced rates of disease-related surgery and lower relapse frequencies.^3,4^ Consequently, achieving mucosal healing has emerged as a pivotal objective in the evaluation of various biologics, including antitumor necrosis factor-α (anti-TNF-α) and ustekinumab (UST)—an anti-IL12/23 antibody.^5–7^ Conversely, the existing data highlight variations in drug effects based on the specific gastrointestinal tract location. Notably, studies have indicated that anti-TNF-α antibodies yield lower rates of mucosal healing in the small intestine than in the large intestine.^8,9^ The precise mechanism underlying the diminished efficacy of anti-TNF-α antibodies in small bowel inflammation versus colorectal inflammation remains elusive. Research has established that attaining mucosal healing through biologics in Crohn’s disease necessitates maintaining a certain trough level.^10–13^ It has been reported that achieving healing in small intestinal inflammation mandates even higher drug blood levels.^14^ These observations suggest potential differences in drug kinetics between the small and large intestines. Additionally, reports underscore variations in cytokine profiles and immune cell dynamics between the 2 intestinal segments in Crohn’s disease, implicating these distinctions in the divergent effectiveness of biological agents.^15–18^ The achievement of mucosal healing in the small intestine not only relates to long-term prognosis but also stands as a crucial therapeutic objective.^2,19^ Despite its paramount importance, a noticeable literature gap exists concerning a direct comparison of biologic efficacy in addressing small intestinal inflammation.^20,21^ In clinical research, the accuracy and utilization of data are essential. In this context, inverse probability weighting (IPW) is superior to propensity score matching (PSM), which can exclude unmatched samples. Inverse probability weighting efficiently estimates outcomes, particularly when matching is tough, and notably reduces selection bias. The adoption of stabilized weighting ensures stability in analysis, even with extreme propensity scores. Inverse probability weighting also balances covariates across groups and adapts to multiple treatments, outpacing traditional PSM. Moreover, it manages missing data, ensuring unbiased results. Given these considerations, our study aimed to bridge the aforementioned knowledge gap by comparing the impacts of anti-TNF-α agents and UST on small intestinal mucosa, while accounting for underlying factors by employing propensity score analysis with IPW.

## Materials and Methods

### Study Design and Patients

We conducted a case–control study aimed at comparing the impacts of anti-TNF-α and anti-IL12/23 antibodies on small intestinal inflammation in Crohn’s disease. Our analysis included 70 adult Crohn’s disease patients who had commenced treatment with either anti-TNF-α or UST between March 2015 and August 2021. These patients had undergone endoscopic evaluation of both small and large intestine mucosa before and 1–2 years after the initiation of antibody therapy.

For the administration of anti-TNF-α agents, the induction protocol for infliximab involved administering an initial dose of 5 mg/kg at weeks 0, 2, and 6, followed by a maintenance dose of 5 mg/kg every 8 weeks. Subcutaneous adalimumab was given at an induction dose of 160 mg initially, followed by 80 mg 2 weeks later, and then a maintenance dose of 40 mg every 2 weeks. In cases where a physician noted reduced efficacy with anti-TNF-α agents, dosage adjustments were implemented as follows: Infliximab could be increased to 10 mg/kg every 8 weeks or administered at 5 mg/kg every 4 weeks, and the single dose of adalimumab could be escalated to 80 mg. Initially, UST was administered as a weight-based intravenous (IV) infusion as part of the induction regimen, followed by a maintenance dosing of 90 mg administered subcutaneously every 8 weeks. Evaluation of the gastrointestinal mucosa involved the utilization of either total colonoscopy or transanal double-balloon endoscopy (DBE). In instances where pretreatment CT scans, small bowel radiography, or MR enterography raised suspicions of small intestinal inflammation, DBE assessments were performed both before and after the treatment. All eligible patients presented with either a small bowel or small bowel-colorectal type of the disease.

The retrospective nature of this study was conducted with the approval of the ethics committee of Osaka Medical and Pharmaceutical College in Japan (Research ID: 2020-112).

### Outcome Measures

For the assessment of Crohn’s disease clinical activity, we employed the Crohn’s Disease Activity Index (CDAI). The CDAI encompasses a comprehensive array of variables, including the number of liquid stools, intensity of abdominal pain, overall well-being, and extraintestinal symptoms, among others. Concurrently, endoscopic evaluation was carried out using the Simple Endoscopic Score for Crohn’s Disease (SES-CD).^22^ The SES-CD score, a widely recognized colonoscopy scoring system for Crohn’s disease, was applied to 5 defined segments, including the terminal ileum. This scoring system entails the assessment of Crohn’s disease activity based on 4 endoscopic criteria: Ulcer size, ulcerated surface, affected surface, and presence of stenosis. In instances where DBE was employed, no cases were observed where the entire small intestine could also be assessed using transoral DBE. Consequently, the evaluation was conducted using SES-CD, rather than modified SES-CD. In this study, we used the notation SES-CD (I) to represent endoscopic scores specific to the small intestine, and SES-CD (C) to denote endoscopic scores exclusive to the colon. A solitary evaluator, blinded to the corresponding clinical outcomes, performed the assignment of endoscopic scores. The evaluation of both the CDAI and SES-CD occurred simultaneously.

### Statistical Analysis

To compare baseline characteristics between treatment groups and evaluate symptom and endoscopic scores pre- and post-treatment, we conducted statistical analyses employing either the paired *t*-test or the chi-square test. To identify factors associated with improvement in small intestinal endoscopic scores, we conducted both univariate regression analysis and multivariate analysis using a generalized linear model. The comparison of small intestinal endoscopic score improvement before and after 1–2 years of administration was performed between the anti-TNF-α antibody administration group and the UST administration group (UST group). This comparison was executed using propensity score analysis with IPW following background matching. The propensity score for each patient was calculated using a logistic regression model. This model incorporated a comprehensive set of covariates believed to influence both treatment decisions and outcomes. These covariates included age, sex, smoking status, body weight, body mass index (BMI), pretreatment concomitant medications (such as steroids, immunomodulators, and elementary diet), history of biologic administration, pretreatment serological markers (albumin [Alb] and C-reactive protein [CRP]), history of bowel resection, presence of coexisting lesions (eg, stenotic or hemorrhoidal lesions), disease characteristics (location and behavior), CDAI at the time of treatment initiation, and SES-CD at induction, as well as disease duration. Stabilized weights were assigned to each patient based on the computed propensity scores.

All statistical analyses were carried out using JMP software (Version 16; SAS Institute, Tokyo, Japan) and Prism (Version 9; GraphPad Software, LLC, Boston, MA). For all analyses, a significance level of *P* < .05 was considered statistically significant. Missing data were not imputed and were excluded from the statistical calculations.

### Ethical Considerations

The study was conducted with the approval of the ethics committee of Osaka Medical and Pharmaceutical College in Japan (Research ID: 2020-112).

## Results

### Patient Characteristics

A total of 70 patients diagnosed with ileal or ileocolonic Crohn’s disease were included in the analysis. Within our cohort of 70 patients, we assessed various characteristics within the anti-TNF-α group (*n* = 47) and the UST group (*n* = 23) (Table 1). The patient distribution was gender-balanced (41.4% females), with a median age of 28 years (IQR 22–40). The predominant disease location was L3 (84.3%), and the most frequent behavior was B1 (54.3%). A history of bowel resection was present in 20% of patients, with stenotic and hemorrhoidal lesions noted in 31.4% and 35.7% of patients, respectively. During induction, steroids were utilized by 34.3% of patients, immunomodulators by 24.3%, and an elemental diet by 60%. A smoking history was reported by 11.4% of patients, and 72.9% were biologically naïve. The median CRP level was 0.97 mg/dL (IQR 0.41–2.06), BMI was 20.59 kg/m^2^ (IQR 18.62–22.31), and albumin (Alb) was 3.4 g/dL (IQR 3.0–4.0). The median CDAI was 161.2 (IQR 123.75–210.1), and the SES-CD was 14 (IQR 8–19). Disease duration had a median of 2.5 years (IQR 1–6.5), and the time until endoscopic efficacy verification was a median of 15 months (IQR 12–21). Notably, significant differences were observed between the 2 groups in terms of the presence of stenotic lesions and the utilization of an elemental diet during induction (*P* = .0387 and *P* = .0209, respectively). Details regarding the patient demographics and clinical characteristics for each therapeutic agent are provided in Table S1.

**Table 1.** Comparison of baseline patient characteristics between the anti-TNF-alpha antibody group and the ustekinumab group.

Characteristics	Overall population (*n* = 70)	Anti-TNF-α group (*n* = 47)	UST group (*n* = 23)	*P* value
Number (%)				
Gender (female) (%)	29 (41.4)	20 (42.6)	9 (39.1)	.7848
Location (L1/L3)	11/59	6/41	5/18	.3326
Behavior (B1/B2/B3)	38/27/5	25/19/3	13/8/2	.918
History of bowel resection	14 (20.0)	9 (19.1)	5 (21.7)	.7991
Presence of stenotic lesions	22 (31.4)	11 (23.4)	11 (47.8)	.0387
Presence of hemorrhoidal lesions	25 (35.7)	17 (36.2)	8 (34.8)	.6349
Steroid use during induction	24 (34.3)	15 (31.9)	9 (39.1)	.5503
Use of immunomodulators during induction	17 (24.3)	13 (27.7)	4 (17.4)	.3467
Use of elementary diet during induction	42 (60.0)	32 (68.1)	10 (43.5)	.0209
Smoking history	8 (11.4)	5 (10.6)	3 (13.0)	.4279
Bio-naïve	51 (72.9)	35 (74.5)	16 (69.6)	.6648
Median (IQR)				
Age (years)	28 (22–40)	27 (22–37)	33 (21–49)	.2733
CRP (mg/dL)	0.97 (0.41–2.06)	1.09 (0.46–1.72)	0.91 (0.34–2.32)	.6931
BMI (kg/m^2^)	20.59 (18.62–22.31)	20.47 (18.46–22.7)	21.24 (20.29–22.08)	.2657
BW (kg)	54.9 (49.45–66.13)	54.3 (49.04–63.99)	56.6 (51.23–67)	.5847
Alb (g/dL)	3.4 (3.0–4.0)	3.4 (2.9–3.9)	3.7 (3.35–4)	.0958
CDAI	161.2 (123.75–210.1)	159.4 (120.8–210.1)	162 (140.5–208.5)	.6214
SES-CD	14 (8–19)	13.5 (7–19)	14 (10.5–17.75)	.7733
SES-CD (I)	4.5 (3–6)	4 (3–6)	5 (3–7.5)	.4841
SES-CD (C)	10.0 (3.25–14.0)	9.5 (3–13.75)	10 (4.5–14)	.9251
Disease duration (years)	2.5 (1–6.5)	2 (1–7)	3 (0.5–5)	.4413
Duration until verification of endoscopic efficacy (months)	15 (12–21)	15.5 (12–21.75)	13 (12–19)	.4337

Data are mean (SD), median (IQR), or *n* (%) unless otherwise stated. *n* = 23 in the UST group and 47 in the anti-TNF-α group.

Abbreviations: Alb, albumin; anti-TNF-α, antitumor necrosis factor-α; BMI, body mass index; CDAI, Crohn’s disease activity index; CRP, C-reactive protein; IQR, interquartile range; SD, standard deviation; SES-CD, Simple endoscopic score for Crohn’s disease; SES-CD (I), SES-CD scores specific to the small intestine; SES-CD (C), SES-CD scores specific to the colon; UST, ustekinumab.

### Effects of Anti-TNF-α and Ustekinumab on Small Intestinal Inflammation

**Figure 1.**
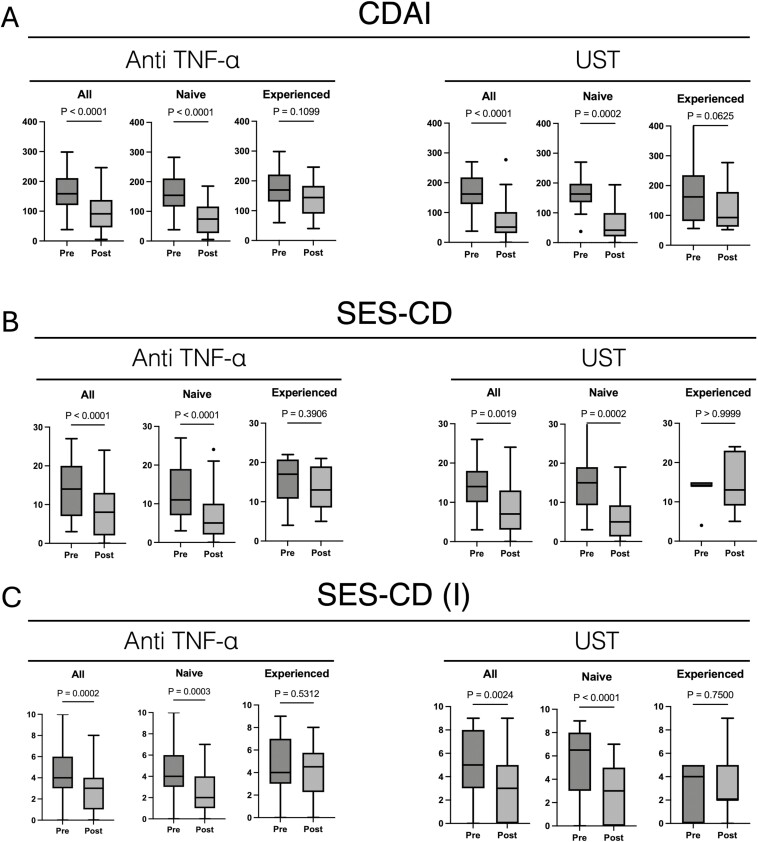
Changes in the clinical and endoscopic scores before and after administration of anti-TNF-α antibody and ustekinumab. (A) CDAI score. (B) Endoscopic score. (C) Small intestine endoscopic score. anti-TNF-α, antitumor necrosis factor-α; CDAI, Crohn’s Disease Activity Index; SES-CD, Simple endoscopic score for Crohn’s disease; UST, ustekinumab.

Clinical symptoms were assessed using the CDAI during the endoscopic evaluation. The results revealed significant improvements in these symptoms within both the anti-TNF-α treatment group (*P* < .0001) and UST treatment group (*P* < .0001; Figure 1A). Furthermore, enhanced efficacy was evident among patients with no previous history of biological agent usage across both treatment groups (Figure 1A). Endoscopic scores, as determined by the SES-CD, exhibited significant post-treatment enhancements in both the anti-TNF-α (*P* < .0001) and UST groups (*P* = .0019), consistent with the clinical symptom outcomes. Notably, patients without a history of biological agent administration demonstrated notably greater improvements (Figure 1B). This trend persisted even when analyses were focused solely on scores from small intestine endoscopy (SES-CD (I); Figure 1C). The small bowel endoscopy score exhibited a marked improvement in ulcer size and area due to the therapeutic intervention (see Figure S1A, which shows the comparison of changes in the small intestine and colon endoscopic scores between both drugs). Conversely, scores for stenosis and lesions other than ulcers did not demonstrate significant changes with the treatment intervention. In the evaluation of nutritional status, a noteworthy enhancement in serum Alb levels was witnessed in both groups before and after treatment (see Figure S2B, which shows improvements in ulcer size and area in both groups). Furthermore, the extent of Alb level improvement exhibited a significant correlation with the improvement in endoscopic scores rather than with the improvement in clinical symptoms (see Figure S2C, which shows the correlation of Alb levels with endoscopic score improvement).

### Multivariate Analysis of Factors Associated With Healing of Small Intestinal Inflammation

**Figure 2.**
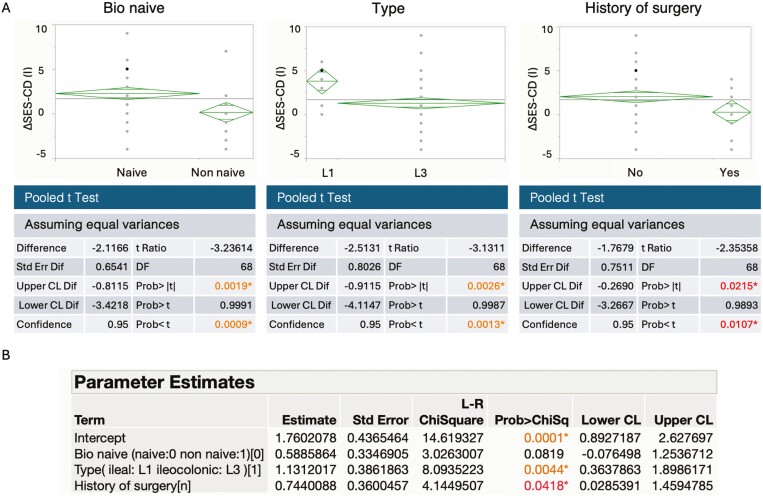
Exploration of factors associated with improvement in small intestine endoscopic scores. (A) Univariate regression analysis for each independent variable against the dependent variable. (B) Multivariate analysis using generalized linear model (GLM).

Subsequently, we delved into the factors contributing to mucosal healing in the small intestine. Our approach entailed a singular regression analysis employing various parameters as explanatory variables, while the degree of improvement in the small intestine’s endoscopic score served as the dependent variable. Among the explanatory variables, those yielding coefficient estimates with *P* values below .20 were considered, and these included no prior use of biologic agents (*P* = .0019), disease type (*P* = .0026), and surgical history (*P* = .0215; Figure 2A). Employing multivariate analysis on the 3 elements identified in the single regression analysis, we found that disease type exhibited a significant correlation with mucosal healing of the small intestine (*P* = .0044; Figure 2B).

### Comparison of the Effects of Anti-TNF-α and Anti-IL-12/23 Antibodies on Small Intestinal Inflammation Using Propensity Score

**Figure 3.**
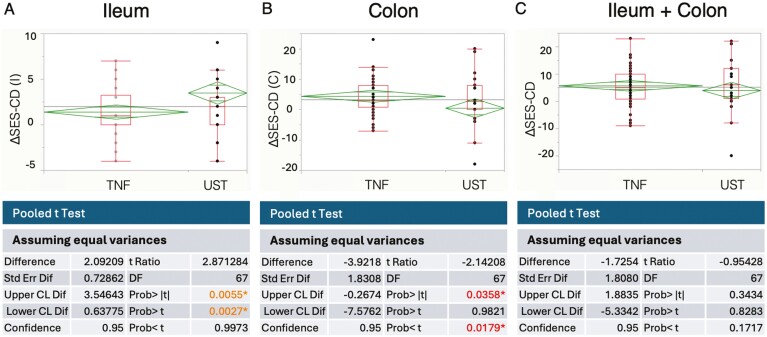
Propensity score analysis using inverse probability weighting (IPW). (A) Small intestine endoscopic scores. (B) Colon endoscopic scores. (C) Overall endoscopic scores. CDAI, Crohn’s Disease Activity Index; TNF-α, tumor necrosis factor-α; SES-CD, Simple endoscopic score for Crohn’s disease; UST, ustekinumab.

Utilizing the technique of IPW to account for baseline characteristics, we conducted a comparison concerning the alteration in small intestine endoscopic scores between the anti-TNF-α and anti-IL12/23 antibody groups. A notably greater degree of improvement emerged in the small intestine endoscopic score in the cohort treated with the anti-IL12/23 antibody than in the anti-TNFα antibody-administered group (*P* = .0055; Figure 3A). In contrast, the extent of improvement in the colon endoscopic score exhibited was higher in the group receiving anti-TNFα antibody (*P* = .0358), while no significant discrepancy was observed in the overall endoscopic score between the 2 groups (*P* = .3434; Figure 3B, C).

## Discussion

Our retrospective observational study provides valuable insights into the comparative effects of anti-TNF-α and anti-IL-12/23 antibodies on small intestinal inflammation in Crohn’s disease patients. By employing propensity scores, our study effectively addressed potential biases arising from background factors. Initially, we examined the improvements in clinical symptoms and endoscopic scores following 1–2 years of treatment with anti-TNF-α and anti-IL12/23 antibody agents. Both classes of agents exhibited significant score improvements, yet the extent of improvement in symptoms and endoscopic findings varied substantially based on the history of biological agent administration. This trend remained consistent with the endoscopic scores of the small intestine. While previously reported data suggested a somewhat weaker effect of anti-TNF-α antibody agents on the small intestine than on the large intestine,^8,9^ the underlying reasons for this discrepancy remain enigmatic. Emerging research, however, suggests the potential efficacy of anti-IL12/23 antibody in treating small bowel inflammation in Crohn’s disease.^23^ Several reports have compared the efficacy and persistence of using anti-TNF-α antibody agents with anti-IL12/23 antibody agents in treating various conditions.^20,24,25^ However, to date, no study has specifically and accurately compared their effectiveness on small intestinal inflammation. Therefore, we aimed to unravel the factors contributing to the improvement in small intestinal endoscopic scores. Multivariate analysis pinpointed disease type, particularly that of the small intestine, as the most significant contributor to the enhancement of small intestinal endoscopic scores (Figure 2). Crohn’s disease is recognized for displaying distinct differences in onset, progression, and risk of complications contingent on the site of inflammation. In our study, we utilized PSM to scrutinize the effects of anti-TNF-α antibody agents and UST on small intestinal inflammation. The analysis revealed that UST exerted a significantly greater improvement effect on small intestinal mucosa (Figure 3). This divergence might be attributed to the fact that the location of inflammation in Crohn’s disease not only influences patient demographics and complications but also yields distinct cytokine profiles. In colonic-type Crohn’s disease, either Th1 or Th17 cytokines are implicated, while the small intestinal type involves a mixed engagement of both Th1 and Th17 cytokines.^26^ Moreover, serum cytokine analyses have reported weaker TNF-alpha expression in small intestinal type than in colonic-type Crohn’s disease.^17^ Furthermore, in newly developed small intestinal inflammation of Crohn’s disease, observations have indicated a mixed Th1/Th17 response without notable TNF-α induction.^27^ This suggests that TNF-α involvement in small intestinal inflammation in Crohn’s disease may be less pronounced than that in colonic inflammation. Furthermore, it is conceivable that UST, capable of inhibiting the Th17 cytokine axis by blocking IL-23, could prove effective in treating small intestinal inflammation in Crohn’s disease. Conversely, in our study, anti-TNF-α agents demonstrated greater efficacy in treating colonic inflammation than UST (Figure 3). This underscores the importance of considering the balance of inflammatory severity in both the small and large intestines when treating Crohn’s disease. Additionally, recent advancements in *in vivo* imaging under endoscopy have enabled the visualization of localized cytokines.^28,29^ Future developments in this area could significantly influence drug selection by enhancing our understanding of the differences in cytokine profiles between the small and large intestines in Crohn’s disease, as well as the cytokine profiles specific to individual cases. Although our study yields crucial insights, certain limitations warrant consideration. First, the retrospective nature of our study could introduce potential biases despite the utilization of propensity score analysis. Second, the study cohort’s relatively small size may limit the generalizability of the findings to larger populations or diverse demographic groups. Moreover, the long-term effects and safety profiles of these treatments were not evaluated in our study.

In conclusion, our study suggests that UST may outperform anti-TNF-α agents in treating small intestinal inflammation in Crohn’s disease. Subsequent prospective studies encompassing larger cohorts are imperative to validate these findings.

## Supplementary Material

otae033_suppl_Supplementary_Material

## Data Availability

Data not publicly available.
