# Supplementary material for: Comparing the Effects of Anti-TNF Agent and Ustekinumab on Small Bowel Inflammation in Crohn’s Disease: Inverse Probability Weighting With Stabilized Weights of Propensity Scores
Source: Crohns Colitis 360. 2024 May 14;6(2):otae033. doi: 10.1093/crocol/otae033 (PMC11165431; doi:10.1093/crocol/otae033)
Supplement: otae033_suppl_Supplementary_Material [file otae033_suppl_supplementary_material.zip › Supplementary_Materials.docx]

SUPPLEMENTARY DATA

Supplementary Data Content 1. Comparison of baseline patient characteristics for each therapeutic agent.

Supplementary Data Content 2. Comparison of the components of endoscopic scores. [A] Comparison of changes in small intestine and colon endoscopic scores between both drugs. [B] Significant improvements in ulcer size and area in both groups: A significant improvement was observed in both ulcer size and area across both groups when comparing the components of endoscopic scores. [C] Correlation of albumin levels with endoscopic score improvement: No correlation was observed between the improvement in albumin levels and clinical symptom scores, while a significant correlation was found with the improvement in endoscopic scores.
