# Supplementary figures and images for: Comparing the Effects of Anti-TNF Agent and Ustekinumab on Small Bowel Inflammation in Crohn’s Disease: Inverse Probability Weighting With Stabilized Weights of Propensity Scores
Source: Crohns Colitis 360. 2024 May 14;6(2):otae033. doi: 10.1093/crocol/otae033 (PMC11165431; doi:10.1093/crocol/otae033)

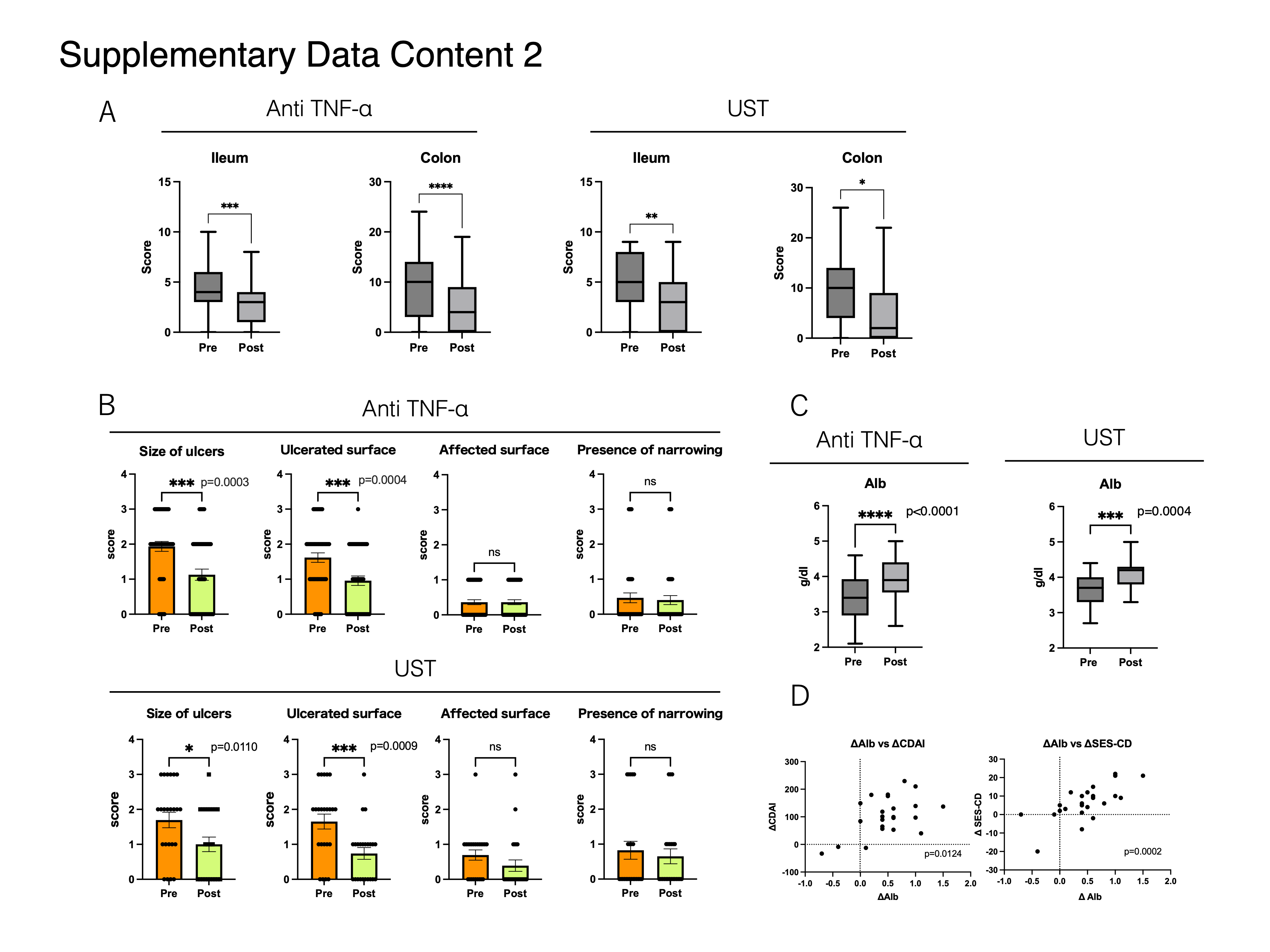

Supplement: otae033_suppl_Supplementary_Material [file otae033_suppl_supplementary_material.zip › Supplementary Data Content2.tiff]
